# Web-Based Cognitive Behavioral Self-Help Intervention to Reduce Cocaine Consumption in Problematic Cocaine Users: Randomized Controlled Trial

**DOI:** 10.2196/jmir.2244

**Published:** 2012-11-28

**Authors:** Michael Schaub, Robin Sullivan, Severin Haug, Lars Stark

**Affiliations:** ^1^Research Institute for Public Health and Addictionassociated to Zurich UniversityZurichSwitzerland; ^2^Mainstation CenterARUDZurichSwitzerland

**Keywords:** Cocaine, Cognitive Behavioral Therapy, Internet, Randomized Controlled Trial

## Abstract

**Background:**

Web-based self-help programs that reduce problematic substance use are able to reach hidden consumer groups in the general population. These programs are characterized by their low treatment threshold and nonrestrictive intervention settings. They are also cost effective, making them of interest to both low-income and high-income industrialized countries with ever-increasing health costs.

**Objective:**

To test the feasibility and effectiveness of an anonymous, fully automated, Web-based self-help intervention as an alternative to outpatient treatment services for cocaine users.

**Methods:**

A total of 196 cocaine-using participants were recruited through various online and offline media for a randomized controlled trial. Participants in the intervention group received interactive cognitive behavioral modules and a consumption diary to reduce cocaine use, whereas participants in the control group received online psychoeducative information modules. Web-based follow-up assessments were conducted after 4 weeks, 6 weeks, and 6 months. Treatment retention was examined and compared between the intervention and control groups. Severity of cocaine dependence was the main outcome measure. Secondary outcomes were cocaine craving, depression symptoms, and alcohol and other substance use.

**Results:**

This Web-based intervention attracted older and more educated participants than existing outpatient treatment programs for which cocaine is the primary substance of abuse. Participants in the intervention group showed greater treatment retention compared with the control group (*P = *.04). Low response rates at the follow-up assessments restricted the explanatory power of the analyses. At the follow-up assessments, the severity of cocaine dependence did not differ between the intervention and control groups (*P = *.75). Furthermore, there were no differences in cocaine craving, depression, or alcohol and other substance use. Using the consumption diaries, the average number of cocaine-free days per week did not change significantly, whereas the weekly quantity of cocaine used decreased equally in both groups (*P = *.009).

**Conclusions:**

For cocaine users with low dependence severity, a fully automated Web-based cognitive behavioral self-help intervention is a feasible alternative with limited effectiveness in outpatient treatment services. However, this type of intervention may attract specific user groups that are rarely reached by existing outpatient treatment and may help them to control their cocaine consumption anonymously.

**Trial Registration:**

ISRCTN93702927; http://www.controlled-trials.com/ISRCTN93702927 (Archived by WebCite at http://www.webcitation.org/6CTMM10MR)

## Introduction

Data on the prevalence of problematic cocaine use and addiction are lacking in Switzerland and many other developed countries, but there is no doubt that cocaine use has increased in Switzerland in recent years [1] and in other Western European countries [2]. In 2005, Swiss resident institutions reported, for the first time in history, that cocaine surpassed opiates as the most frequently abused substance [3]. This trend has also been observed in outpatient units [3]. This increase in treatment requests likely reflects only a minority of cocaine users. Typically, those cocaine users in outpatient treatment who do not report co-consumption of opiates are young and have low education levels [3]. Older and more educated cocaine users, who are likely to be better integrated into society, are rarely reached by standard treatment. Presumably, the majority of these individuals consume cocaine on a quasi-controlled basis and only a small fraction of them are likely to take advantage of treatment [4]. However, it is likely that some of these users will progress from controlled use to problematic use [5]. For more educated and integrated cocaine users, anonymous interventions that follow the principle of concurrent cover (ie, noninvasive, low-cost interventions in which therapeutic intensity can be enhanced and extended to face-to-face treatment according to need) appear to be more appropriate. Thus, the diversification of the available outpatient treatment services for cocaine users in this direction is favorable.

Over the past 12 years, a number of interventions enhanced by information and communication technology (ICT) have aimed to optimize various aspects of mental health care, such as the treatment of eating disorders [6], obesity [7], depression [8], and social phobia [9]. The majority of these approaches have been based on Internet and mobile phone technologies, such as text messaging [10]. Web-based self-help programs that reduce problematic consumption are able to reach hidden consumer groups in the general population because of their low treatment threshold and nonrestrictive setting for intervention [11]. Furthermore, these programs show a remarkably positive cost-benefit relation [12], which is of interest in industrialized countries with widespread Internet access and escalating health costs. These programs have been tested primarily in people with tobacco dependence or problematic alcohol use. The existing reviews and meta-analyses of Web-based interventions for tobacco smoking and alcohol use [13-16] show that these interventions are superior to no or minimal intervention; however, the effect sizes that have been reported are predominantly small. Evidence concerning their effectiveness compared with face-to-face interventions is inconclusive [13,17,18]. To date, few studies exist on the effectiveness of Web-based interventions for the treatment of illegal substance use. In a controlled trial, a Web-based intervention designed to help young people quit or reduce their cannabis use was tested [18]. Despite some methodological constraints, the results of this study showed that Web-based intervention is promising in the reduction of cannabis consumption compared to no intervention. To date, no research on the acceptance and effectiveness of a Web-based program for the treatment of problematic cocaine use has been conducted.

Snow Control, a 6-week Internet-based self-help intervention program for problematic cocaine users who intend to control, reduce, or stop their consumption of cocaine, was tested between March 2010 and December 2011 and compared with a control condition in a randomized controlled trial [19]. The treatment aim was moderation of cocaine use or cocaine abstinence, with participants in the Snow Control intervention group expected to show greater reductions in cocaine consumption after 6 weeks of treatment than the control participants. Moreover, we hypothesized that the participants in the intervention group would show greater improvements at the 6-week treatment termination point in secondary outcomes, including (1) cocaine craving, (2) alcohol intake, (3) use of illicit substances other than cocaine, and (4) symptoms of depression. We also anticipated the participants in the intervention group to show significantly greater retention. Overall, we aimed to test the feasibility and effectiveness of an anonymous, fully automated, Web-based self-help intervention as an alternative to outpatient treatment services for cocaine users.

## Methods

### Interventions

Snow Control is based on cognitive behavioral therapy (CBT) methods that have been tested on cocaine addicts [20,21], principles of motivational interviewing [22], current self-control practices, and the established relapse-prevention model [23-25].

The intervention is structured into 8 modules that are activated for week-by-week access in the first 3 weeks, with 4 additional voluntary modules that can be activated during weeks 4 to 6. A detailed description of the intervention can be viewed in the study protocol [19] (trial registration ISRCTN93702927). After successful registration, participants were randomized by computer program in a 1:1 ratio to 1 of 2 parallel groups. Participants were blinded to the interventions. After the first week in the intervention group, each log-in directed the participant to his or her consumption diary in which he or she was asked to specify, for each day, the amount of cocaine consumed in the past 7 days and the amount of cocaine he or she planned to consume each day for the next 7 days. The participant was then directed to the respective weekly module.

To assess the effectiveness of the Snow Control intervention, an appropriate psychoeducative online control condition was developed. Participants in the control condition received 8 psychoeducative information modules about risks, potential harm, and other important information about cocaine consumption followed by a quiz to evaluate their knowledge. The duration of the control condition was equal to the 6 weeks of the experimental intervention; however, the control condition did not include the whole consumption diary. Participants in the control condition were asked to specify the amount of cocaine consumed in the previous 7 days, but not the amount of cocaine they planned to consume in the next 7 days.

To avoid serious harm to the participants in the intervention and control condition during the study, a detailed consent procedure with thorough safety instructions was provided as well as a continuously accessible 24-hour emergency list (including the numbers of emergency help lines and contact information for the study team and the webmaster), regardless of whether participants withdrew or dropped out of the study. Moreover, during the 6-week intervention phase, the participants had the opportunity to contact a corresponding outpatient clinic in a nearby city by telephone (lists with opening hours, Web links, postal addresses, and telephone numbers were provided).

### Measurement Instruments

All outcome measures were assessed through online questionnaires. After providing informed consent, participants who met the study entry criteria created a personal and secure log-in name and password and received an automated email notification with their access information. They were then directed to a baseline assessment Web page with questions regarding sociodemographic characteristics and consumption patterns. The primary outcome measures of cocaine consumption were recorded as the number of days and quantity of cocaine used, in milligrams, as specified in the consumption diary and reflected by the Severity of Dependence Scale (SDS) [26] score. The secondary outcomes consisted of the following: (1) the Cocaine Craving Questionnaire-Brief (CCQ-Brief) [27], (2) selected measures for the assessment of the past month’s consumption and method of consumption for *Diagnostic and Statistical Manual of Mental Disorders* (Fourth Edition)/*International Classification of Diseases, Tenth Revision* (*DSM-IV*/*ICD-10*) substances of abuse derived from the European version of the Addiction Severity Index (EuropASI) [28], and (3) a short German version of the Beck Depression Inventory (BDI) [29]. In addition, we asked participants to provide feedback about any technical and substance use problems during the intervention. We assessed the qualitative feedback after 6 weeks of intervention. We also planned to explore participants’ use of cocaine and other substances at a 6-month follow-up. Because we expected the follow-up rates to be low, compensation (€40) was offered to participants who logged in and completed the follow-up questionnaires.

### Analyses

Generalized estimating equation (GEE) analyses were carried out to investigate the effectiveness of the intervention on different variables assessed at baseline and various follow-up points over the study period of 6 months. The GEE is a repeated-measures regression model that takes into account the correlation between the repeated measures of each person [30]. We performed logistic GEE analyses for the binary outcome variables and linear GEE analysis for continuous outcome variables. An alpha level of .05 (2-tailed) was chosen for all statistical tests in this study. Due to the low response rate at the follow-up assessments, we applied multiple regression imputation methods to impute missing data on the investigated variables using the imputation by chained equations (ICE) procedure of Stata’s statistical software [31]. We applied the intention-to-treat principle and considered all randomized participants in the analyses. We crosschecked our results by running the analyses with the nonimputed dataset.

History data were analyzed with descriptive statistics and general linear models for repeated measures using group membership as a between-subject factor. Because retention was crucial in this study, we explored the baseline predictors of 6-week retention, defined as completion of the consumption diary, using binary logistic regression analyses. First, all potential predictor variables were entered into a preliminary regression model. Next, variables that were not significant (*P *≥ .05) were systematically removed; only variables that were significant (*P < *.05) were retained in the model.

### Recruitment

The study participants were recruited between March 2010 and October 2011 through the Snow Control website; websites of outpatient treatment centers in the Canton of Zurich, Switzerland; websites of national organizations for alcohol and drug prevention in nightlife settings; and tailored advertisements on national social media platforms. In addition, advertisements were placed on national Internet forums, newspapers, and on 2 television reports that were broadcasted on Swiss Television. People interested in participating received more information on the Snow Control website. The website explained the rationale of the study, the different assessments, assessment schedules, and the assessment duration. The participants were informed about (1) study inclusion and exclusion criteria, (2) the potential risks of participation, (3) safety arrangements during and after the study phase, (4) the inability of Snow Control to replace face-to-face therapy for problematic cocaine use/abuse, and (5) the circumstances under which they should contact their general practitioner or a professional from the medical advisory and emergency list that was made accessible at all times and how to make this contact. The participants were also informed that the study was reviewed by the ethics committee of the Canton of Zurich and given their declaration of no objection (*nihil obstat*). Moreover, they were informed about their right to withdraw from the study at any time without consequences. Informed consent was accepted when participants clicked on a field on the informed consent page and submitted the consent with a submission button.

The study inclusion criteria were a minimal age of 18 years and cocaine use on at least 3 occasions in the past 30 days. The exclusion criteria consisted of participation in other psychosocial or pharmacological treatments for the moderation or cessation of cocaine use, reports of opioid use in the past 30 days (with the exception of substitution maintenance treatment for opioid dependence without street heroin use in the last 30 days), and previous treatment for cardiovascular problems or apoplexy. The exclusion criterion of a BDI score > 55 was omitted because the average BDI depression characteristics were above the 55-point score.

**Figure 1:**
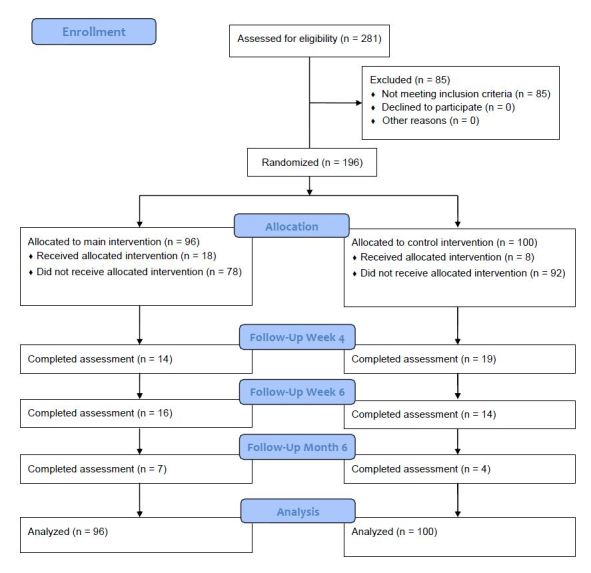
Flowchart of study participants.

The flow of study participants is depicted in Figure 1. A total of 281 participants successfully registered online, provided their informed consent, and completed the baseline assessment, but 85 (30.2%) did not meet the following eligibility criteria: (1) age ≥ 18 and (2) cocaine use on at least three occasions in the last 30 days [19]. Consequently, these participants were excluded from further analyses. A total of 69 participants (24.6%) reported less than 3 days of cocaine consumption in the past 30 days (n = 31 in intervention group and n = 38 in control group), but 7 of these participants (2.5%) reported frequent use of amphetamines and began using Snow Control to control their amphetamine use (n = 5 in intervention group and n = 2 in control group). Another 8 participants (2.8%) who were not excluded for other reasons reported street heroin use in the past 30 days (n = 3 in intervention group and n = 5 in control group); and 8 participants (2.8%) were currently being treated for cardiovascular diseases (n = 3 in intervention group and n = 5 in control group). Therefore, 196 participants who met the inclusion criteria entered the study and were randomly allocated to the intervention or control conditions using the background database. Participants who were not randomized because they did not meet the inclusion criteria were allowed to participate in the intervention. Recruitment ended after the intended number of participants in the study protocol was exceeded (n = 196).

## Results

### Baseline Characteristics

There were no differences between the Snow Control intervention group and the control group in the examined baseline variables (Table 1). Compared with the participants whose main substance problem was cocaine who entered Swiss outpatient addiction treatment (n = 429) during 2010, the participants in this study (n = 196) were older (Chi-square [χ^2^_1_] = 3.3, *P = *.001, Cohen’s effect size w [w] = 0.132) and more educated (university degree: 11.7 vs 3.9; higher professional education: 23.5 vs 6.4; high school degree: 15.8 vs 11.6; apprenticeship/vocational school: 39.8 vs 67.2; and obligatory school: 9.2 vs 12.0, χ^2^_1_ = 6.6, *P = *.001, w = 0.196).

**Table 1:** Baseline characteristics of the participants in the Snow Control (intervention) group and control group.

Characteristics		Snow Control	Control group	Total	*t* test ^a^	Chi-square^a^
		(n = 96)	(n = 100)	(N = 196)	(*t*_194_)	(χ^2^_1_)
**Gender n (%)**						
	Female	22 (22.9)	21 (21.0)	43 (21.9)		0.3
	Male	74 (77.1)	79 (79.0)	153 (78.1)		
Age, mean (SD)		34.9 (9.1)	33.4 (8.5)	34.2 (8.8)	1.150	
**Highest degree of education, n (%)**						
	Obligatory school	7 (7.3)	11 (11.0)	18 (9.2)		0.5
	Apprenticeship, vocational school	39 (40.6)	39 (39.0)	78 (39.8)		
	High school degree	15 (15.6)	16 (16.0)	31 (15.8)		
	Higher professional education degree	24 (25.0)	22 (22.0)	46 (23.5)		
	University degree	11 (11.5)	12 (12.0)	23 (11.7)		
**Questionnaire scores, mean (SD)**						
	Severity of Dependence Scale (SDS)	7.8 (3.3)	8.2 (3.0)	8.0 (3.1)	1.006	
	Cocaine Craving Questionnaire-Brief (CCQ-Brief)	44.3 (9.8)	43.9 (10.6)	44.1 (10.3)	0.095	
	Beck Depression Inventory (BDI)	55.5 (12.6)	57.7 (14.9)	56.6 (13.9)	1.309	
Years of cocaine consumption, mean (SD)		6.2 (6.2)	7.2 (7.5)	6.7 (6.9)	0.992	
**Method of cocaine consumption (multiple answers possible), n (%)**						
	Nasal	86 (89.6)	96 (96.0)	182 (92.9)		0.1
	Smoked	14 (14.6)	12 (12.0)	26 (13.3)		1.7
	Oral	11 (11.5)	12 (12.0)	23 (11.7)		0.5
	Injected (nonintravenous)	1 (1.0)	4 (4.0)	5 (2.6)		1.3
	Injected (intravenous)	3 (3.1)	2 (2.0)	5 (2.6)		0.6
**Lifetime substance use, n (%)**						
	Amphetamines, ecstasy	20 (20.8)	27 (27.0)	47 (24.0)		1.1
	Cannabis	53 (55.2)	59 (59.0)	112 (57.1)		0.8
	Benzodiazepines, barbiturates	11 (11.5)	7 (7.0)	18 (9.2)		1.1
	Heroin	4 (4.2)	4 (4.0)	8 (4.1)		0.1
	Methadone	3 (3.1)	1 (1.0)	4 (2.0)		1.0
**Treatment for addiction-related problems during lifetime, n (%)**		19 (19.8)	21 (21.0)	40 (20.4)		0.0
**Substance use at least once last 30 days before baseline assessment, n (%)**						
	Amphetamines, ecstasy	18 (18.7)	19 (19.0)	27 (18.9)		0.0
	Cannabis	37 (38.5)	49 (49.0)	86 (43.9)		1.8
	Benzodiazepines, barbiturates	14 (14.6)	8 (8.0)	22 (11.2)		1.4
	Heroin	0 (0)	0 (0)	0 (0)		—
	Methadone	2 (2.1)	2 (2.0)	4 (2.0)		1.0
	Alcohol use	82 (85.4)	86 (86.0)	168 (85.7)		0.6
	Binge alcohol use	40 (41.7)	36 (36.0)	80 (40.8)		0.6

^a^ None of the comparisons was significant (*P* ≤ .05).

The participants in this study reported an average of 6.7 years (SD 6.9) of cocaine use and their most frequent method of use was snorting cocaine (182/196, 92.9%). Most of the participants had not used heroin (188/196, 95.9%) or methadone (192/196, 98.0%) in their lifetimes. The use of amphetamines or ecstasy, substances typically consumed during local nightlife activities [23], was reported by 47 (24.0%) participants. Of the included participants, 54 (27.6%) had been previously treated for a depression disorder, 20 (10.2%) had been treated for an anxiety disorder, and 12 (6.1%) had been treated for other diseases, such as attention deficit disorder/attention deficit hyperactivity disorder (6/196, 3.0%), anorexia (2/196, 1.0%), psychosis (3/196, 1.5%), and borderline personality disorder (1/196, 0.5%). A total of 8 participants (4.1%) reported being positive for the human immunodeficiency virus. The relevant baseline variables did not differ between groups (Table 1). In addition, the intervention and control groups did not differ with respect to the receipt of treatment for addiction-related problems (χ^2^_1_ = 0.0, *P = *.83, w = 0) or mental health-related problems (depression: χ^2^_1_ = 0.2, *P = *.63, w = 0.001; anxiety: χ^2^_1_ = 0.7, *P = *.40, w = 0.004).

### Intervention Participation

Participants in the Snow Control intervention group completed more modules (mean 2.60, SD 2.04) than those in the control group (mean 1.80, SD 1.60; *t*_194_ = 3.086, *P = *.002, Cohen’s d [d] = 0.438). Because intervention modules were accessible week-by-week in both groups, this result also reflects the average time, in weeks, that participants remained in their intervention. Overall, the average number of days that elapsed between the first and last log-ins did not differ between the intervention group (mean 32.53, SD 31.52) and the control group (mean 27.44, SD 20.53; *t*_194_ = 1.335, *P = *.28, d = 0.191).

### Retention

**Figure 2:**
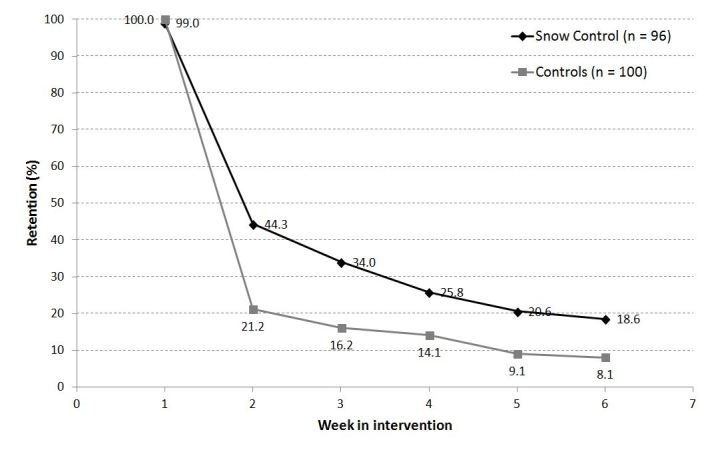
Retention in the Snow Control online self-help intervention (n = 96) and the control condition (n = 100).

According to the consumption diary data, retention in the intervention group (see Figure 2) was significantly greater than that of the control group (week 6: χ^2^_1_ = 2.1, *P = *.04, w = 0.220). The contact rate for questionnaires at the 6-month follow-up was very low for both the Snow Control intervention group (7/96, 7.3%) and the control group (4/100, 4%).

Table 2 depicts the final predictor model for treatment retention. Inclusion in the intervention group was related to retention at week 6 (odds ratio [OR] = 2.65, CI 1.04 - 6.77, *P = *.04). Other relevant factors were age (OR = 1.05, CI 1.01 - 1.10, *P = *.047) and depression symptoms (OR = 1.06, CI 1.02 - 1.11, *P = *.004). The severity of cocaine dependence was associated with treatment retention and was below 1.0 (OR = 0.76, CI 0.64 - 0.92, *P = *.005) indicating that participants with higher severity scores showed poorer treatment retention.

**Table 2:** Logistic regression of baseline variables for retention at Week 6.

Variables	Odds ratio (95% CI)	*P*
Condition (0 = control group, 1 = intervention group)	2.65 (1.04-6.77)	.04
Age (range 18-56)	1.05 (1.01-1.10)	.047
Severity of dependence (SDS, range 1-10)	0.76 (0.64-0.92)	.004
Depressive symptoms (BDI, range 20-91)	1.06 (1.02-1.11)	.005

### Study Outcome Results

Table 3 presents the results of the GEE analyses using the imputed dataset for continuous outcomes. As seen in Table 4, no significant group × time interactions in the severity of cocaine dependence (*P = *.75) were seen. Furthermore, no significant group × time interactions were observed in the secondary outcomes of cocaine craving (*P = *.90) or depression (*P = *.57).

Table 5 presents the results of the GEE analyses using the imputed dataset for binary outcomes.

As seen in Table 6, no group × time interactions were found between in the consumption of cannabis, cocaine, or alcohol at the follow-up assessments (P ≥ .05). We observed similar results for the GEE analyses of the nonimputed dataset, which resulted in no significant group × time interaction terms for any of the investigated variables.

**Table 3:** Descriptive statistics of the continuous outcome variables from the imputed dataset.

Continuous outcome variables		Baseline	4 weeks	6 weeks	6 months
**Severity of Dependence Scale, mean (SD)**							
	Intervention group	7.8 (3.3)	7.3 (5.2)	5.2 (3.4)	3.8 (2.1)
	Control group	8.2 (3.0)	7.1 (5.3)	5.4 (3.4)	4.0 (2.2)
**Cocaine Craving Questionnaire-Brief, mean (SD)**							
	Intervention group	44.3 (9.8)	46.3 (12.1)	48.5 (11.2)	47.4 (7.2)
	Control group	43.9 (10.6)	45.1 (14.1)	47.8 (11.4)	46.6 (7.6)
**Beck Depression Inventory, mean (SD)**							
	Intervention group	55.5 (12.6)	—	51.8 (16.3)	45.0 (10.5)
	Control group	57.7 (14.9)	—	54.3 (16.9)	45.6 (10.6)

**Table 4:** Results from linear generalized estimating equation (GEE) models examining the effect of study group (control group vs Snow Control intervention), time, and study group × time interaction terms on cocaine dependence, cocaine craving, and depression.

Continuous outcome variables		Beta	Standard error	*t* test	*P*
**Severity of Dependence Scale (SDS)^a^ (degrees of freedom [df] = 8.4)**					
	Study group (control vs intervention)	–0.36	0.74	–0.49	.63
	Time	–1.45	0.23	–6.25	.000
	Study group × time	0.07	0.22	0.33	.75
**Cocaine Craving Questionnaire-Brief (CCQ-Brief)^a^ (df = 7.3)**					
	Study group (control vs intervention)	0.67	1.92	0.35	.73
	Time	1.07	0.78	1.37	.21
	Study group × time	0.07	0.60	0.12	.90
**Beck Depression Inventory (BDI)^b^ (df = 6.5)**					
	Study group (control vs intervention)	–2.86	2.69	–1.06	.29
	Time	–4.45	1.09	–4.08	.006
	Study group × time	0.43	0.76	0.57	.57

^a^ Variable was assessed at baseline, and at 4-week, 6-week, and 6-month follow-ups.

^b^ Variable was assessed at baseline, and at 6-week and 6-month follow-ups.

**Table 5:** Descriptive statistics of the binary outcome variables from the imputed dataset.

Binary outcome variables		Baseline	4 weeks	6 weeks	6 months
**Cannabis consumption within previous month, %**					
	Intervention group	38.5	57.1	66.7	84.0
	Control group	48.6	60.2	69.4	89.2
**Cocaine consumption within previous month, %**					
	Intervention group	100	75.8	71.2	66.9
	Control group	100	76.6	76.4	62.4
**Alcohol consumption within previous month, %**					
	Intervention group	85.4	73.5	76.9	100
	Control group	86.0	74.4	77.2	100
**Binge drinking within previous month, %**					
	Intervention group	41.7	60.2	52.5	74.2
	Control group	36.0	62.8	59.6	71.4

**Table 6:** Results from logistic generalized estimating equation (GEE) models examining the effect of the study group (control group vs Snow Control intervention), time, and the study group × time interaction terms on the consumption of different substances.

Binary outcome variables^a^		OR (95% CI)^b^	Standard error	*t* test	*P*
Cannabis consumption within previous month (df = 9.4)					
	Study group (control vs intervention)	0.73 (0.30 - 1.79)	0.33	–0.69	.49
	Time	1.92 (1.32 - 2.79)	0.32	3.93	.003
	Study group × time	1.03 (0.72 - 1.47)	0.18	0.18	.86
Cocaine consumption within previous month (df = 4.0)					
	Study group (control vs intervention)	0.78 (0.17 - 3.55)	0.22	–0.07	.95
	Time	0.42 (0.07 - 2.50)	0.45	–0.58	.59
	Study group × time	1.08 (0.70 - 1.67)	0.24	0.36	.72
Alcohol consumption within previous month (df = 10.0)					
	Study group (control vs intervention)	0.95 (0.42 - 2.15)	0.39	–0.13	.89
	Time	1.42 (1.06 - 1.90)	0.19	2.61	.02
	Study group × time	1.01 (0.74 - 1.37)	0.15	0.07	.95
Binge drinking within previous month (df = 5.0)					
	Study group (control vs intervention)	1.13 (0.45 - 2.84)	0.52	0.28	.78
	Time	1.57 (0.77 - 3.21)	0.44	1.64	.16
	Study group × time	0.94 (0.66 - 1.34)	0.16	–0.35	.73

^a^ Variables were assessed at baseline, and at 4-week, 6-week, and 6-month follow-ups.

^b^ OR: odds ratio.

### Consumption Diaries

**Figure 3:**
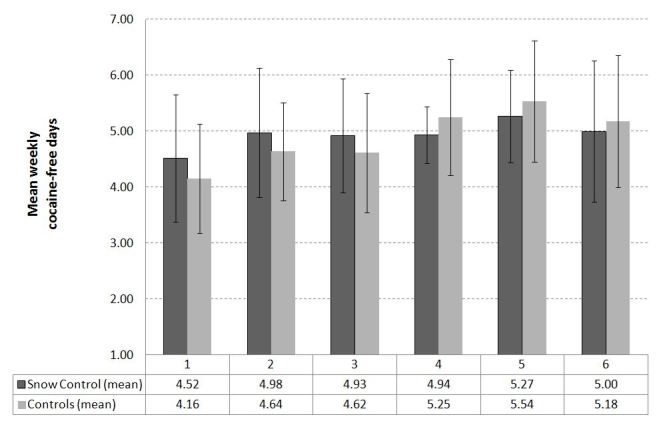
Mean weekly cocaine-free days for weeks 1 to 6.

**Figure 4:**
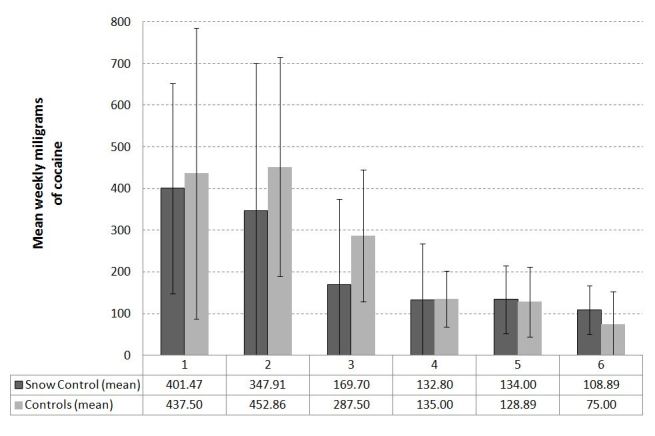
Mean weekly milligrams of cocaine for weeks 1 to 6.

According to the consumption diaries (see Figure 3), there were no differences in the reduction of the average weekly use of cocaine (in milligrams) between the intervention and control groups at week 4 (*t*_37_ = 0.077, *P *=.94, d = 0.010) or week 6 (*t*_24_ = 0.544, *P = *.59, d = 0.245). Similarly, the mean number of cocaine-free days per week recorded in the consumption diaries did not differ between groups at week 4 (*t*_46_ = 2.225, *P = *.31, d = 0.512) or week 6 (*t*_30_ = 0.841, *P = *.94, d = 0.079). Overall, the average number of cocaine-free days per week did not change significantly between week 1 and 6 (*t*_31_ = -1.189, *P = *.24, d = 0.311, imputed data set: *t*_195_ = 1.26, *P = *.21), whereas the weekly quantity of cocaine used decreased equally in both groups between week 1 and 6 (*t*_25_ = 3.188, *P = *.004, d = 0.761, imputed data set: *t*_195_ = 2.63, *P = *.009) as seen in Figure 4.

### Adverse Events and Additional Help

During the study, 13 participants (6 in Snow Control intervention and 7 in control group) contacted outpatient treatment services for additional help as indicated on the website. Of these participants, 5 received medical advice by telephone (3 in Snow Control intervention and 2 in control group), and 8 (3 in Snow Control intervention and 5 in control group) entered an outpatient treatment service because they found the help received through the website to be insufficient. Most of these participants reported impulsive cocaine use and/or severe psychiatric comorbidity.

## Discussion

According to the results of our study, the implementation of a fully automated cognitive behavioral online self-help intervention for the reduction of cocaine use is feasible, but of limited effectiveness when compared with a psychoeducative active control condition in a sample of relatively treatment-naive cocaine users. There was not a greater improvement in the severity of cocaine dependence in the Snow Control intervention group than in the control group. Participants in the intervention group who remained in treatment reduced their average weekly use of cocaine (in milligrams) to a similar level as that observed in the control group; the average weekly cocaine-free days were somewhat higher in the control group, but did not change substantially in either group. Cocaine craving, alcohol use, binge drinking, use of illicit substances other than cocaine, and depression characteristics also did not improve compared with controls. Study retention and intervention participation were higher in the Snow Control intervention group, suggesting that this type of intervention was more attractive to participants than the alternative psychoeducative information, corresponding quiz, and limited consumption diary that was presented to the control group.

One reason that only very small differences were observed between the intervention and control group might lie in the comparable durations for each module and the similar stepwise weekly access to the modules. Sessions for both groups were designed to demand similar time from their users [19], and a short consumption diary was even implemented in the control condition to ensure comparability. Thus, a significantly greater reduction in cocaine consumption in the Snow Control intervention would have reflected the superiority of a fully automated cognitive behavioral self-help intervention to an active control condition.

One obvious reason why we did not find a greater reduction in the frequency of cocaine use or in the severity of cocaine dependence was the fact that the majority of participants chose to reduce the quantity of cocaine consumed, but did not choose to increase the number of cocaine-free days. This finding was the case although we communicated that this intervention was intended to help participants control or reduce cocaine use or to achieve cocaine abstinence [19]. Thus, the users of the Snow Control intervention focused on moderation of cocaine use and prevented the weekly escalation of cocaine use by controlling their quantity when using, but not by increasing their number of cocaine-free days. In other words, they followed a harm-reduction strategy.

## Limitations

Although the number of questionnaires was limited, the participants demonstrated a clear aversion to completing the questionnaires. This aversion was the primary flaw in the study design. Many participants filled out the consumption diary and used the designed modules or read the psychoeducative texts, but they simply closed their Internet browsers when the questionnaires began. The implementation of telephone contact to increase study retention, as performed in similar studies for the reduction of alcohol [14] or tobacco use [13], was clearly rejected in the pilot study [19] because cocaine users may fear repressive activities by the police or other authorities. Furthermore, the compensation (€40) for the follow-up assessment did not motivate the participants to log in again and complete the questionnaires. A similar compensation at week 6 was not possible due to budget constraints and it was not initially included in the study protocol [19].

The dropout rates for completion of the consumption diary (81.2% in the intervention and 92% in the control group) were higher than we expected (70%) when we designed the study as a randomized controlled trial. In addition to inclusion in the intervention group, factors that contributed to the retention of participants in treatment until week 6 included the low severity of symptoms of cocaine dependence, age, and depression symptoms, suggesting that the online self-help format is difficult to follow for more severely cocaine-dependent participants and has better retention for depressed and older cocaine users.

Future variations of the intervention will attempt to increase retention by implementing personal, but anonymous, chat contacts similar to those implemented in an online self-help intervention for cannabis users [32]. Additionally, the integration of Snow Control into a national addiction online counseling portal in which it will be possible to compare this self-help intervention with professional online counseling by email contacts is planned. Future studies will integrate modules addressing depression symptoms and will attempt to prevent users from failing to complete the evaluation questionnaires.

We strongly recommend the development of a consumption diary as the primary outcome measure for Internet-based studies aimed at the reduction of illicit substance use. Additionally, if feasible, contingency management (compensation for online-intervention attendance) might increase treatment retention. Unfortunately, in addition to the financial limitations of this study, this contingency management strategy was not feasible in this study in Switzerland due to the structure of the treatment supply center and the probable strong rejection from health authorities and politics.

### Conclusions

We conclude that a fully automated Web-based cognitive behavioral self-help intervention is feasible, but of limited effectiveness compared with a psychoeducative control group for cocaine users with low dependence severity. This type of intervention may attract older and more educated participants than existing outpatient treatments for which cocaine is the primary substance of abuse and might help to control participants’ cocaine consumption. Future studies should attempt to improve treatment retention through additional Web-based approaches, such as anonymous chat sessions, and investigate the program’s effectiveness in more detail.

## Multimedia Appendix 1

CONSORT Ehealth Checklist V1.6 [33].

## Abbreviations

BDI: Beck Depression Inventory
CBT: cognitive behavioral therapy
χ2: Chi-square test
CCQ-Brief: brief version of the Cocaine Craving Questionnaire
d: Cohen’s d
DSM-IV: Diagnostic and Statistical Manual of Mental Disorders (Fourth Edition)
EuropASI: European version of the Addiction Severity Index
GEE: generalized estimating equation
ICD-10: International Classification of Diseases, Tenth Revision
ICE: imputation by chained equations
ICT: information and communication technology
OR: odds ratio
SDS: Severity of Dependence Scale
t: t test
w: Cohen’s effect size w
